# The official soundtrack to “Five shades of grey”: Generalization in multimodal distractor-based retrieval

**DOI:** 10.3758/s13414-020-02057-4

**Published:** 2020-06-12

**Authors:** Lars-Michael Schöpper, Tarini Singh, Christian Frings

**Affiliations:** 1 Department of Cognitive Psychology, University of Trier, Trier, Germany; 2 Department of Experimental Psychology, Martin-Luther-University Halle-Wittenberg, Halle (Saale), Germany

**Keywords:** Action control, Attention, Stimulus-response binding, Perception

## Abstract

When responding to two events in a sequence, the repetition or change of stimuli and the accompanying response can benefit or interfere with response execution: Full repetition leads to benefits in performance while partial repetition leads to costs. Additionally, even distractor stimuli can be integrated with a response, and can, upon repetition, lead to benefits or interference. Recently it has been suggested that not only identical, but also perceptually similar distractors retrieve a previous response (Singh et al., *Attention, Perception, & Psychophysics, 78*(8), 2307-2312, 2016): Participants discriminated four visual shapes appearing in five different shades of grey, the latter being irrelevant for task execution. Exact distractor repetitions yielded the strongest distractor-based retrieval effect, which decreased with increasing dissimilarity between shades of grey. In the current study, we expand these findings by conceptually replicating Singh et al. (2016) using multimodal stimuli. In Experiment 1 (N=31), participants discriminated four visual targets accompanied by five auditory distractors. In Experiment 2 (N=32), participants discriminated four auditory targets accompanied by five visual distractors. We replicated the generalization of distractor-based retrieval – that is, the distractor-based retrieval effect decreased with increasing distractor-dissimilarity. These results not only show that generalization in distractor-based retrieval occurs in multimodal feature processing, but also that these processes can occur for distractors perceived in a different modality to that of the target.

## Introduction

Humans constantly interact with their environment. A closer look at actions shows that for actually executing an action several cognitive modules have to work in concert. The resulting cognitive processes and the mechanisms that contribute to the cognitive part of the action are still an area of intense research. In particular, it has been demonstrated that the selection, activation, and initiation of actions is influenced by binding and memory processes linking actions to the stimuli that previously accompanied the execution of this action, and to the effects that it has produced in the past. These processes are at the heart of action control research (see, e.g., Frings, Hommel, et al., 2020, for a recent framework on the processes underlying human action control), and there are several theories that explain how actions are actually executed. For instance, according to the theory of event coding, when responding to a stimulus, the stimulus features and the response are integrated into a temporary episodic memory trace, known as an *event file* (Hommel, 1998, 2004; Hommel, Müsseler, Aschersleben, & Prinz, 2001). Such an event file can be investigated in prime-probe sequences in which participants react to a prime target followed by a probe target. When all stimulus and response features from the prime are repeated in the probe, the prime event file is retrieved, leading to faster reaction times and lower error rates (as the retrieved response is compatible to the probe response). If the feature configurations and the response are only partially repeated, the previous event file is retrieved; however, since it does not completely match the current event, it interferes with the current response execution. Such *stimulus-response bindings* are ubiquitous in many sequential paradigms (e.g., Henson, Eckstein, Waszack, Frings, & Horner, 2014; Frings, Hommel, et al., 2020; Frings, Koch, et al., 2020) and are believed to underlie many simple actions (Frings, Hommel, et al., 2020; Frings, Koch, et al., 2020; however, see, e.g., Schöpper, Hilchey, Lappe, & Frings, in press, for the absence of such binding effects in detection performance).

Not only relevant but also irrelevant stimulus features are bound to a response, in so-called *distractor-response bindings* (DRB; Frings, Rothermund, & Wentura, 2007). If a distractor (or a distractor feature) is bound to a response, this can cause benefits or interference on subsequent trials: when both the response and the distractor are repeated (response repetition, distractor repetition; RRDR), benefits occur, because the previous information is retrieved, and it matches the current event. When only the distractor repeats, but the required response changes (response change, distractor repetition; RCDR), the distractor retrieves the previous event-file; however, since the retrieved event file contains inappropriate response information, the retrieval causes interference. Similarly, when the response repeats, but the distractor changes (response repetition, distractor change; RRDC), the repeated response retrieves the previous event file, which does not match the current event, causing interference. In this condition, however, since the distractor is changed, there is no distractor-based retrieval. When both response and distractor change (response change, distractor change; RCDC), there is no interference because nothing is retrieved from the previous event-file.

According to Giesen and Rothermund (2014), the DRB paradigm shows some structural similarities to Pavlovian conditioning (Pavlov, 1927). In the famous example of Pavlovian conditioning (e.g., Mazur, 2006), the dog starts to produce a salivary response or reflex (unconditioned response) on hearing a bell (conditioned stimulus) that signals the presence of food (unconditioned stimulus). Giesen and Rothermund (2014) reasoned that the distractor in the DRB paradigm can be seen as the conditioned stimulus, with the target and response being the unconditioned stimulus and response, respectively: if the distractor (conditioned stimulus) is presented along with the target (unconditioned stimulus), it is associated with the response to the target (unconditioned response). Thus, upon a subsequent presentation of the distractor, the (associated) response to the target is executed. With these thoughts in mind, Singh, Moeller, and Frings (2016) hypothesized that processes that impact Pavlovian conditioning should also impact DRB effects: Because in Pavlovian conditioning generalization takes place, that is, conditioned responses can occur in situations that are similar, but not exactly the same as those situations they were learned in (Pearce, 1987), generalization might take place in distractor-based retrieval as well.

To investigate this, Singh et al. (2016) had participants discriminate between different stimuli that demanded different responses based on their shape. Crucially, the shapes appeared in one of five shades of grey only differing in their lightness values and fully irrelevant for task execution. From prime to probe, the lightness value could repeat or deviate with increasing difference in the lightness values, resulting in five different distractor relations. In exact repetition trials, the exact same lightness value repeated from prime to probe. In three trial types with lightness value deviation, lightness values could deviate from prime to probe in three increasing degrees of dissimilarity. The fourth and most noticeable lightness value deviation from prime to probe was labelled the *distractor change*. For each distractor condition a DRB effect was calculated as (RRDC-RRDR)-(RCDC-RCDR) for reaction times and error rates, while using the largest lightness value difference as the distractor change condition for all four distractor repetition conditions with exact repetition and decreasing similarity as the repetition conditions. In line with the generalization processes observed in Pavlovian conditioning (Pearce, 1987), the DRB effect was largest for the exact distractor repetition condition and decreased with increasing distractor dissimilarity; that is, Singh et al. (2016) observed generalization processes in distractor-based retrieval (see Fig. 2A for the original data).

Taking the example of Pavlovian conditioning further, if the bell is rung in the field of vision of the dog, it seems plausible that the visual information of the bell (shape, color, movement, etc.) might be conditioned as well. The conditioned stimulus thus can be defined by several modalities or as a combination of these – namely, as a multimodal stimulus. Event files can include not only visual features but also features of other modalities, like audition (e.g., Spence, & Frings, 2020; Zmigrod & Hommel, 2009), even if the auditory information is irrelevant (e.g., Moeller, Rothermund, & Frings, 2012; for a review, see Frings, Schneider, & Moeller, 2014). Crucially, event files that involve combinations of different modalities, like vision, audition, or touch – and thus being multimodal – have been observed (for a review, see Zmigrod & Hommel, 2013). For example, Zmigrod, Spapé, and Hommel (2009; Experiment 1) used a design similar to Hommel (1998) in which participants saw two circles in one of two colors accompanied by a tone in one of two pitches in a sequence. The response to the first target was cued by a left/right cue and the second response was a discrimination response based on the identity of the second target. In one task participants had to discriminate the color of the second stimulus, whereas in the other task, they had to discriminate the pitch of the second stimulus. In both tasks they found a binding pattern congruent with the idea of multimodal event-files, that is, binding between auditory and visual information with the response. In conclusion, a distractor presented in a modality different from the modality the target is perceived with, is capable of retrieving a previous event file (see also Frings et al., 2014). Thus, the generalization in distractor-based retrieval found by Singh et al. (2016) should also occur for distractors presented in a different modality.

### The present study

In the current study we conceptually replicated the design of Singh et al. (2016), but presented the targets and distractors in different modalities. In Experiment 1, visual targets were accompanied by auditory distractors, whereas in Experiment 2 auditory targets were accompanied by visual distractors. This design allows us not only to replicate Singh et al. (2016), but – by using targets and distractors perceived with different modalities and reversing these from Experiment 1 to Experiment 2 – to expand their findings to a generalization of distractor-based retrieval in multimodal stimulus-response bindings.

## Methods

### Experiment 1

In Experiment 1 we investigated if distractor-generalization takes place in a modality irrelevant for task execution by presenting visual targets accompanied by auditory distractors. Procedure and all materials closely resemble and conceptually replicate Singh et al. (2016). If generalization of distractor-based retrieval occurs in multimodal stimulus-response bindings, we should observe a data pattern comparable to Singh et al. (2016).

#### Participants

We used the same sample-size as in Singh et al. (2016). Thirty-one students (20 women, 11 men, *M*_*age*_ = 22.32 years, *SD*_*age*_ = 5.12 years, age range: 18–47 years) from the University of Trier participated for either course credit or voluntarily.

#### Design

The experimental design used a 2 (response relation: repetition vs. change) x 5 (distractor relation: exact repetition vs. repetition with one step deviation vs. repetition with two steps deviation vs. repetition with three steps deviation vs. change), all varied within-subjects.

#### Apparatus and materials

The experiment was run with E-Prime Software Version 2.0 on a computer screen with a display resolution of 1,680 x 1,050 px. Four shapes (square, diamond, cross, and triangle) appeared at the center on the screen completely in black on a white background (as there were no color differences in grey scale, we did not use the black-and-white striped background used in Singh et al., 2016). Participants sat approximately 60 cm in front of the computer screen, resulting in stimulus sizes^1^ of approximately 2.39° x 2.39° of visual angle and the black fixation cross appearing in 0.29° x 0.29° of visual angle. As distractors, we used sine wave tones with five different frequencies (400 Hz, 420 Hz, 440 Hz, 460 Hz, 480 Hz), which were created using Audacity (Audacity Team), resulting in five different pitches. The distractors were presented via headsets (Creative Labs Fatal1ty HS-800 Gaming Headset) with on average 62.8 dB (with a slight increase of loudness with increasing frequency from 61.4 dB to 64.2 dB) measured on one side of the headphones using a XL2 Audio and Acoustic Analyzer with M4260 microphone (NTi Audio; Schaan, Liechtenstein).

#### Procedure

Participants sat approximately 60 cm in front of the screen wearing headphones. The instructions appeared on-screen. All stimuli and the fixation cross appeared in the center of the screen. A trial started with the onset of a fixation cross for 1,000 ms. The fixation cross was followed by the presentation of the prime display, that is, the target shape and the distractor sound. The auditory distractor accompanied the visual target for 200 ms; however, the visual target remained on-screen until response. Participants were instructed to respond to a diamond or a triangle by pressing the F-key, and to a cross or square by pressing the J-key.^2^ After the prime response, the screen turned blank for 500 ms followed by the probe display. The structure of the probe display was identical to the prime display. After a response to the probe was detected, the next trial started. The experiment was divided into 32 practice trials and 600 experimental trials. For practice trials, participants received positive or negative feedback after every response. For experimental trials, participants only received feedback after incorrect responses; in such cases, an error message appeared for 1,500 ms. After half the trials, participants were allowed to take a self-paced break.

**Fig. 1:**
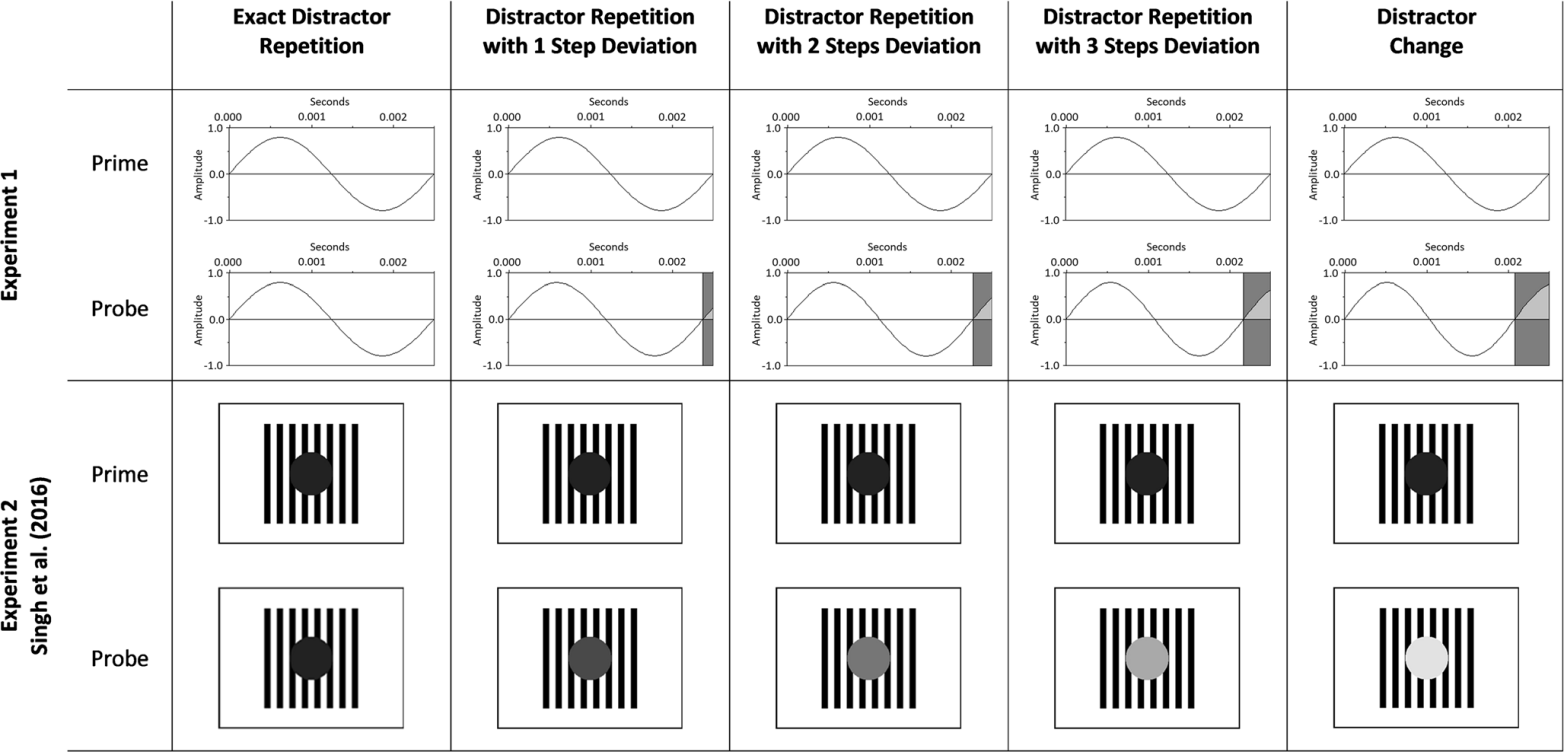
Example distractor relations of Experiment 1 (**top panel**), and Experiment 2 and Singh, Moeller, and Frings (2016) (**bottom panel**). In exact distractor repetition trials, the same distractor repeated from prime to probe. In distractor repetitions with 1 step deviation, the distractor deviated 20 units (Experiment 1: in Hz; Experiment 2: in *L*) above or below the prime distractor. In distractor repetitions with 2 steps deviation and 3 steps deviation, the distractor deviated 40 units and 60 units, respectively. In distractor change trials, the distractor had the largest deviation of 80 units. Note that prime-probe sequences could start with any of the distractors. Top panel: Visualization of the sine waves that were used to generate the sounds (see main text). The frequency (Hz) of a sine wave is the number of oscillations per second; the duration of one oscillation, that is the period (*T*), can be calculated by *T* = 1/Hz. For example, a sine wave with 400 Hz has a period of 0.0025 seconds. With increasing frequency, the duration of a period gets shorter and the pitch increases. Bottom panel: In Experiment 2 and Singh et al. (2016) five different shades of grey varying in lightness values were used as distractors

In response-repetition (RR) trials, participants responded with the same key for both prime and probe. As there were always two shapes matched to one key, response repetition trials could be due to shape repetition or due to a shape mapped to the same key press. In response-change (RC) trials, participants responded with a different key from prime to probe, that is, a shape indicating a different key appeared in the probe display. In distractor-repetition (DR) trials, the tone frequency could either be exactly repeated (exact repetition) or was repeated with varying degrees of similarity (see Fig. 1, top panel). Trials in which the tone frequency repeated with a frequency deviation of 20 Hz above or below the previous distractor were most similar (one step deviation), followed by a larger deviation of 40 Hz (two steps deviation), and 60 Hz (three steps deviation). Trials that had a deviation of 80 Hz were considered as distractor-change (DC) trials. Note that these were simply the most distinct and dissimilar distractor deviations (i.e., four steps deviation). To avoid participants’ performance being affected by different distractor probabilities, we balanced the occurrence of all distractor frequency combinations across all trials. As a result, for example, what is labelled as distractor change can only be observed for a small number of distractor combinations, whereas one-step deviations occur more often. All in all, ten combinations of response relation x distractor relation were possible, resulting in a total of 60 trials of exact repetition, 96 trials of one step deviation, 72 trials of two steps deviation, 48 trials of three steps deviation, and 24 trials of distractor change, for response repetition and response change, respectively.

### Results

#### Reaction times

Trials were only included if the responses to the prime and probe display were both correct. Only probe reaction times that were above 200 ms or below 1.5 interquartile range above the third quartile of a participant’s distribution (Tukey, 1977) were included in the analysis. Due to these constraints, 13.17% of probe trials were excluded from analysis.

**Fig. 2:**
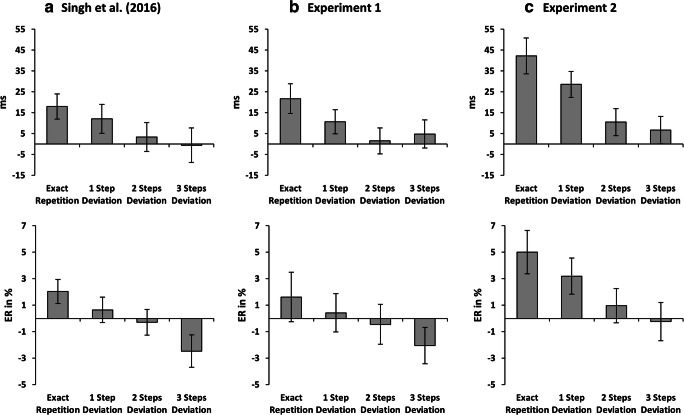
Distractor-response binding effects calculated with reaction times in ms (**top panel**) and error rates in percentages (**bottom panel**) for each of the four distractor repetition conditions, separate for (**A**) Singh et al. (2016), (**B**) Experiment 1, and (**C**) Experiment 2. Error bars represent standard error

We performed a 2 (response relation: repeated vs. changed) x 5 (distractor relation: exact repetition vs. repetition with one step deviation vs. repetition with two steps deviation vs. repetition with three steps deviation vs. change) repeated-measures MANOVA on probe reaction times. There was a main effect of response relation, *F*(1, 30) = 36.56, *p* < .001, ${\upeta}_p^2$ = .55, with participants reacting faster when a response repeated (473 ms) compared to when it changed (511 ms). There was no main effect of distractor relation, *F*(4, 27) = 1.25, *p* = .315, ${\upeta}_p^2$ = .16. Importantly, we found an interaction of response relation x distractor relation, *F*(4, 27) = 4.92, *p* = .004, ${\upeta}_p^2$ = .42 (see Table 1, left panel). This interaction showed a benefit for exact distractor repetition, when the response repeated, but that this benefit decreased with decreasing similarity between prime and probe distractor. In contrast, higher distractor similarity caused more interference in response-change trials. As in Singh et al. (2016) we calculated the DRB effects for each distractor condition to pinpoint this interaction. A DRB effect can be calculated as (RRDC-RRDR)-(RCDC-RCDR); we used the DC condition (i.e., a frequency dissimilarity of 80 Hz) as the DC conditions for all four DR conditions.^3^ We performed a single-factor repeated-measures MANOVA^4^ on the four calculated DRB effects (see Fig. 2B, top panel) using the strength of distractor deviation as the only factor. The main effect of decreasing similarity was significant, *F*(3, 28) = 6.10, *p* = .003, ${\upeta}_p^2$ = .40, in that the DRB effect was largest when the distractor exactly repeated, but became smaller with increasing deviation. The linear trend of this decrease was significant, *F*(1, 30) = 14.24, *p* = .001, ${\upeta}_p^2$ = .32.

**Table 1:** Mean reaction times (in ms) of Experiment 1 and Experiment 2, separate for Response Repetition (RR) and Response Change (RC), as well as all distractor conditions

	Experiment 1		Experiment 2	
	RR	RC	RR	RC
Distractor repetition				
Exact repetition	464	515	415	485
1 step deviation	472	513	424	479
2 steps deviation	478	510	436	474
3 steps deviation	474	509	441	475
Distractor change	478	508	447	474

#### Error rates

The error rate was calculated as the percentage of incorrect probe responses after correct prime responses; that is, trials were only included in the analysis if the prime response was correct. Due to these constraints, 4.94 % of trials were excluded from analysis.

We performed a 2 (response relation: repeated vs. changed) x 5 (distractor relation: exact repetition vs. repetition with one step deviation vs. repetition with two steps deviation vs. repetition with three steps deviation vs. change) repeated-measures MANOVA on probe error rates. Neither the main effect of response relation, *F*(1, 30) = 2.57, *p* = .120, ${\upeta}_p^2$ = .08, nor the main effect of distractor relation, *F*(4, 27) = 1.64, *p* = .192, ${\upeta}_p^2$ = .20, was significant. Crucially, we found an interaction of response relation x distractor relation, *F*(4, 27) = 4.00, *p* = .011, ${\upeta}_p^2$ = .37 (see Table 2, left panel). As with the reaction times, a single-factor repeated-measures MANOVA^5^ on the calculated DRB effects (see Fig. 2B, bottom panel) revealed a significant main effect of decreasing similarity, *F*(3, 28) = 4.36, *p* = .012, ${\upeta}_p^2$ = .32. Again, the linear trend was significant, *F*(1, 30) = 11.36, *p* = .002, ${\upeta}_p^2$ = .28.

**Table 2:** Mean error rates in percentages of Experiment 1 and Experiment 2, separate for Response Repetition (RR) and Response Change (RC), as well as all distractor conditions

	Experiment 1		Experiment 2	
	RR	RC	RR	RC
Distractor repetition				
Exact repetition	3.52	6.31	3.15	8.45
1 step deviation	3.81	5.40	3.67	7.17
2 steps deviation	3.82	4.55	4.62	5.89
3 steps deviation	4.47	3.58	4.33	4.39
Distractor change	3.73	4.90	4.20	4.50

#### Discussion

In Experiment 1, we investigated generalization processes in multimodal distractor-based retrieval by conceptually replicating Singh et al. (2016). Whereas Singh et al. (2016) used visual targets and visual distractors, we combined two modalities: targets were presented visually, but were accompanied by auditory distractors with varying degrees of similarity. Crucially, we fully replicated the data pattern observed by Singh et al. (2016): auditory distractors were integrated when responding to visual targets and caused benefits or interference when later on repeated, depending upon whether the response was repeated or changed. This binding effect was strongest if the distractor was exactly repeated but decreased with an increase of distractor deviation.

One might argue that the distractor deviations used in Experiment 1 are not clear-cut (in the sense of *equal-loudness contours*, e.g., Fletcher & Munson, 1933; Robinson & Dadson, 1956): for example, the frequency shift of 20 units from 400 Hz to 420 Hz might be perceived as weaker (in loudness) than the shift from 460 Hz to 480 Hz. However, we argue that a more fine-tuned selection of sound frequencies – deviations being discrete by acknowledging (subjective) human sound perception versus our selection of deviations as being technically discrete by a frequency change of 20 Hz – would only have accentuated the linear trend we already observed.

Our main hypothesis is that distractor-based retrieval is influenced by generalization processes irrespective of the modality involved. One might argue that auditory stimuli are hard to ignore (Spence, Ranson, & Driver, 2000), cause alertness when used as a cue (Fernandez-Duque & Posner, 1997) or even if unattended (Van der Lubbe & Postma, 2005), and are distracting (e.g., if novel or deviant from a standard tone; Escera, Alho, Winkler, & Näätänen, 1998); a binding pattern might have resulted because the auditory information, although irrelevant for task execution, always had to be involuntarily processed by the cognitive system when computing a response. To address this, we conducted a second experiment, in which we reversed the target- and distractor-defining modalities: In Experiment 2, participants discriminated auditory targets while viewing visual distractors. If distractor-based retrieval processes function the same irrespective of the modality involved in processing the distractor, Experiment 2 should yield a binding pattern comparable to Experiment 1.

## Experiment 2

In Experiment 2, we presented auditory targets accompanied by five visual distractors. Procedure and all materials closely resembled Singh et al. (2016) and Experiment 1. However, to ensure that participants did not close their eyes during the experiment, we instructed the participants to fixate the center of the screen and included 40 catch trials (see below).

### Participants

Thirty-two students (22 women, 10 men, *M*_*age*_ = 22.03 years, *SD*_*age*_ = 2.09 years, age range: 18–28 years) from the University of Trier participated for either course credit or voluntarily. One participant had previously participated in Experiment 1.

### Design, apparatus, and materials

Design, apparatus, and materials were the same as for Experiment 1, except for the following. Two shapes (circle and square) appeared in five different lightness values of the color grey (10, 30, 50, 70, and 90 of the *L* value in the LAB color space with *a* and *b* values at zero, see Fig. 1, bottom panel; these grey values were used by Singh et al., 2016). Contrary to Experiment 1, but as in Singh et al. (2016), both shapes (each 2.39° x 2.39° of visual angle) were presented on a black-and-white striped background with a visible size of 5.25° x 5.53° (length x height; the image itself was 5.53° x 5.53° but ended on the left and right side with a white stripe) of visual angle in front of the white background. With every shape, an auditory stimulus appeared in one of four different frequencies, that is, 400 Hz (in 61.5 dB; dB measured as reported for Experiment 1), 420 Hz (in 61.9 dB), 600 Hz (in 70.5 dB), and 620 Hz (in 71.0 dB). Through this, lower and higher sounds were clearly distinct by frequency, but also slightly distinct by loudness.

### Procedure

Procedure was as described for Experiment 1, except for the following. Participants were instructed to always focus on the center of the screen, because a visual stimulus onset would signal the start of the next trial. Additionally, they were instructed that in a few trials a square would appear, which forbade a response (see below). Target stimuli were presented with headphones. A target in a prime or probe was a sound that appeared for 200 ms and was accompanied by a visual distractor stimulus in greyscale. The visual distractor remained on-screen until a response was given. Participants were instructed to respond to the lower pitches (400 Hz and 420 Hz) by pressing the F-key, and to the higher pitches (600 Hz and 620 Hz) by pressing the J-key. To ensure that participants could tell them apart, the four sounds were presented in isolation and demanded the correct key press to continue as part of the instructions. To avoid the possibility that participants fully ignore the visual display, we included 40 catch trials, in which a square appeared either in the prime (20 trials) or in the probe (20 trials) display with one of the four sounds. In both cases the square was equally likely to appear in any of the five shades of grey, and the accompanying sound was equally likely to be of a low or a high pitch. However, participants were instructed to not press any button when a square was presented, but simply wait; in such a catch trial, the next display started after 1,000 ms. The experiment was divided into 32 practice trials and 640 experimental trials, the latter including the 600 trial combinations as described for Experiment 1 as well as 40 catch trials.

### Results

We excluded all catch trials for analysis, that is, all prime-probe sequences that either involved a square in the prime or in the probe. Three participants had less than 50 % accuracy in catch trials; however, excluding these participants had no influence on the overall results, so they were included in the analysis.

#### Reaction times

We used the same cut-off criteria as reported for Experiment 1. Due to these constraints, 15.75 % of trials were excluded from analysis.

We performed a 2 (response relation) x 5 (distractor relation) repeated-measures MANOVA on probe reaction times. There was a main effect of response relation, *F*(1, 31) = 63.05, *p* < .001, ${\upeta}_p^2$ = .67 (response repetition: 433 ms; response change: 477 ms) and a main effect of distractor relation, *F*(4, 28) = 5.79, *p* = .002, ${\upeta}_p^2$ = .45 (exact repetition: 450 ms; one step deviation: 452 ms; two steps deviation: 455 ms; three steps deviation: 458 ms; change: 461 ms). Importantly, we found an interaction of response relation x distractor relation, *F*(4, 28) = 7.14, *p* < .001, ${\upeta}_p^2$ = .51 (see Table 1, right panel). A single-factor repeated-measures MANOVA^6^ on the calculated DRB effects (see Fig. 2C, top panel) revealed a significant main effect of decreasing similarity, *F*(3, 29) = 8.42, *p* < .001, ${\upeta}_p^2$ = .47. As with Experiment 1, this linear trend was significant, *F*(1, 31) = 26.54, *p* < .001, ${\upeta}_p^2$ = .46.

#### Error rates

We used the same inclusion criteria as reported for Experiment 1. Due to these constraints, 6.90 % of trials were excluded from analysis.

We performed a 2 (response relation) x 5 (distractor relation) repeated-measures MANOVA on probe error rates. There was a main effect of response relation, *F*(1, 31) = 11.20, *p* = .002, ${\upeta}_p^2$ = .27 (response repetition: 3.99 %; response change: 6.08 %) and a main effect of distractor relation, *F*(4, 28) = 3.69, *p* = .015, ${\upeta}_p^2$ = .35 (exact repetition: 5.80 %; one step deviation: 5.42 %; two steps deviation: 5.26 %; three steps deviation: 4.36 %; change: 4.35 %). Again, we found an interaction of response relation x distractor relation, *F*(4, 28) = 5.72, *p* = .002, ${\upeta}_p^2$ = .45 (see Table 2, right panel). A single-factor repeated-measures MANOVA^7^ on the calculated DRB effects (see Fig. 2C, bottom panel) revealed a significant main effect of decreasing similarity, *F*(3, 29) = 7.87, *p* = .001, ${\upeta}_p^2$ = .45. Again, the linear trend was significant, *F*(1, 31) = 21.23, *p* < .001, ${\upeta}_p^2$ = .41.

### Discussion

In Experiment 2 we again found the generalization of distractor-based retrieval effects, that is, an exact repetition of a shade of grey yielded the strongest DRB effect and decreased with decreasing similarity. Crucially, by using visual distractors and auditory targets, we multi-modally replicated Singh et al. (2016) and cross-modally replicated Experiment 1. To pinpoint that the underlying generalization processes are analogue, that is, irrespective of being caused by auditory distractors (Experiment 1) or visual distractors (Experiment 2) when responding to the opposing modality, respectively, we decided to statistically compare the DRB effects of both experiments.

### Comparison between tasks

We analyzed the four DRB effects as a within-subjects factor and included experiment as a between-subjects factor. Excluding the data of Experiment 2 of the participant who had participated in both of the experiments (see *Participants* section of Experiment 2) did not change the overall interpretation of the results, so we decided to include all data.

#### Reaction times

For probe reaction times we performed a 4 (strength of distractor deviation: DRB with exact distractor repetition vs. DRB with one step deviation vs. DRB with two steps deviation vs. DRB with three steps deviation) repeated-measures MANOVA with Experiment (Experiment 1 vs. Experiment 2) as a between-subjects factor.

As expected, there was a main effect of distractor deviation, *F*(3, 59) = 13.47, *p* < .001, ${\upeta}_p^2$ = .41, depicting the decrease of the DRB effect depending on distractor dissimilarity (DRB with exact distractor repetition: 32 ms; DRB with one step deviation: 20 ms; DRB with two steps deviation: 6 ms; DRB with three steps deviation: 6 ms). The main effect of experiment was not significant, *F*(1, 61) = 2.22, *p* = .142, ${\upeta}_p^2$ = .04. Importantly, the interaction between distractor deviation and experiment was not significant, *F*(3, 59) = 1.86, *p* = .146, ${\upeta}_p^2$ = .09.

#### Error rates

For probe error rates we performed the same analysis as for the reaction times, that is, a 4 (strength of distractor deviation: DRB with exact distractor repetition vs. DRB with one step deviation vs. DRB with two steps deviation vs. DRB with three steps deviation) repeated-measures MANOVA with Experiment (Experiment 1 vs. Experiment 2) as a between-subjects factor.

Again, there was a main effect of distractor deviation, *F*(3, 59) = 10.36, *p* < .001, ${\upeta}_p^2$ = .35 (DRB with exact distractor repetition: 3.31 %; DRB with one step deviation: 1.81 %; DRB with two steps deviation: 0.26 %; DRB with three steps deviation: -1.15 %). The main effect of experiment was not significant, *F*(1, 61) = 1.51, *p* = .224, ${\upeta}_p^2$ = .02. Again, the interaction between distractor deviation and experiment was not significant, *F*(3, 59) = 0.58, *p* = .631, ${\upeta}_p^2$ = .03.

### Discussion

The cross-modal analysis between experiments revealed that the generalization of distractor-based retrieval in stimulus-response episodes functions irrespective of which modality constitutes the distractor when responding to a target in a different modality.

## General discussion

In two experiments we investigated whether distractor similarity leads to retrieval effects in prime-probe sequences when the distractor is perceived in a different modality than the target. For this we used the design of Singh et al. (2016), who found that DRB effects are strongest for exact distractor repetitions but decrease with increasing distractor dissimilarity. We fully replicated those findings: an exact distractor repetition led to a benefit when repeating a response; however, this benefit decreased with increasing distractor dissimilarity. On the other hand, an exact distractor repetition led to interference when changing a response and this interference decreased with increasing distractor dissimilarity. Importantly, this type of generalization of distractor-based retrieval was observed irrespective of which modality constituted the distractor when responding to a target in a different modality. In a follow-up comparison we could show that at least as far as vision and audition are concerned the modality does not matter. As with Singh et al. (2016), the current findings can be interpreted as generalization processes comparable to those observed in Pavlovian conditioning (Pearce, 1987).

One might argue that by including catch trials in Experiment 2, which were indicated by the presentation of a square, processing of visual information was necessary to find out when not to respond.^8^ Furthermore, in Experiment 2 participants were instructed that the onset of a visual stimulus would indicate the start of the next trial. Thus, the visual modality in Experiment 2 was not completely task irrelevant compared to the auditory modality in Experiment 1. However, the color of the circle or square could be any shade of grey, and thus what we refer to as the distractor (i.e., the color) was still completely irrelevant for task execution.

The present results along with previous studies (Giesen & Rothermund, 2014; Singh et al., 2016) would indeed indicate some kind of similarity between the distractor-binding task and Pavlovian conditioning. Similar to the pairing of auditory and visual stimulus information in the present experiments, Pavlovian conditioning can also result from learning compounds consisting of, for example, auditory and visual information (e.g., Rescorla, 1988; Rescorla & Wagner, 1972).^9^ Congruent with that, it has been postulated that short-term bindings may be a first step in long-term learning (e.g., Dutzi & Hommel, 2009; Frings, Hommel, et al., 2020; Wolfensteller & Ruge, 2011), particularly as there is a striking resemblance in the procedural details of both paradigms. In this regard, the idea that some kind of feature overlap between episodes leads to retrieval of “past” information influencing current action can also be directly referred to the learning literature. In particular, already according to Estes (1950), a stimulus and a response to it consist of a finite number of environmental events through which occurrence learning takes place; crucially, participants also learn to respond when only a subset of these events repeat (that could be interpreted as feature overlap). Transferring this to the current study, in exact distractor-repetition trials, the highest number of stimulus events repeated. This number of repeated events decreased with increasing distractor deviations, yielding interference for response repetitions (i.e., a smaller subset of events demanding the same response), but benefits for response changes (i.e., a smaller subset of previously learned events not interfering with the other response).

However, the precise interaction between short-term bindings and learning and the possible role that short-term associations may have in longer-term learning are still not completely clear. On the one hand, if a distractor is already associated with a specific response through long-term learning processes, an incompatible distractor will not be associated with that response (Moeller & Frings, 2014). Moreover, Moeller and Frings (2017a) observed that automatic and overlearned stimulus-response associations hinder short-term bindings. On the other hand, Colzato, Raffone, and Hommel (2006; Experiment 3) found no significant differences in the binding effects for familiar shape-color combinations (e.g., a yellow banana) and unfamiliar shape-color combinations (e.g., a red banana). Finally, Moeller and Frings (2017b) showed that short-term binding and long-term learning are two separate processes. However, the present results underscore the similarities between short-term bindings and long-term associations that have previously been postulated and observed, that is, structural similarities (Giesen & Rothermund, 2014) and generalization processes (Denkinger & Koutstaal, 2009; Singh et al., 2016).

In fact, this is not the first study to show generalization processes in auditory stimulus perception. Mondor, Hurlburt, and Thorne (2003; Experiment 2) were interested in whether the cost of response repetitions without target repetition would increase with increasing target dissimilarity. The authors used a prime-probe design, in which participants responded to a frequency that could exactly repeat in the probe (labeled as *identical* trials), increasingly deviate but still demanding the same response (labeled as *equivalent* trials), or switch to a frequency demanding the other response (labeled as *different* trials).^10^ In their experiment, a prime sound of either a low (500 Hz) or a high (3000 Hz) frequency was presented, each demanding a certain key press. This was followed by a probe sound: For each prime frequency, probe frequency could be either identical or deviate with a small, medium, or large deviation above or below the prime frequency (or the frequency changed to demanding the other key). Participants’ performance was best for *identical* trials, but worse in *equivalent* trials, that is, when repeating the response with a deviating target. Importantly, performance got worse with increasing frequency dissimilarity. Mondor et al. (2003) – although emphasizing that this interpretation is speculative – assumed that participants have a tendency to repeat a response when a stimulus repeats, but a tendency to change a response when a stimulus changes (in the sense of a *bypass rule*; see, e.g., Fletcher & Rabbitt, 1978; Krueger & Shapiro, 1981): In *equivalent* trials, a changing stimulus demanding the previous response would cause a tendency to change the response, setting in re-evaluation processes of stimulus-response mappings, which would cause more interference for larger deviations. However, especially in the light of the current study, the results can also be explained in the sense of stimulus-response bindings (e.g., Frings, Hommel, et al., 2020; Hommel, 2004), that is, a probe stimulus (and response) retrieving the prime stimulus (and response) (see also Frings et al., 2014; for a discussion of how binding effects can be better explained by retrieval processes and not by the *bypass rule* see, e.g., Frings et al., 2007): If a probe stimulus slightly deviates from a prime stimulus, performance gets worse and declines with higher probe deviation, because the retrieved event file increasingly mismatches on a perceptual level. Note, however, that in Mondor et al. (2003) the manipulated frequency deviations were task-relevant, in contrast to our use as distractors in Experiment 1.

More generally, previous research already found that visual and auditory features can be integrated together (Jordan, Clark, & Mitroff, 2010), form multimodal event files (Zmigrod et al., 2009; Zmigrod & Hommel, 2013), and are subject to distractor-based retrieval effects (Frings et al., 2014). Here we suggest that the resulting multimodal event files are – when integrated and retrieved – subject to generalization processes as have been previously observed in the visual modality. Taken together, our results support the interpretation of Singh et al. (2016), that “S-R bindings are not just simple associations between a specific stimulus and specific response; rather, they appear to be structured bindings involving multiple levels of representation of responses, stimuli and tasks” (p. 2311). In the current study we show that these multiple levels involve (multiple) representations of stimuli as perceived by different modalities. Furthermore, this means that not only the exact same target stimulus with certain irrelevant features is bound to a response, but also that these irrelevant features can retrieve the previous response without the target stimulus. This even occurs if the irrelevant feature is perceptually different, but similar.

### Conclusion

In two experiments we could show that distractors perceived in a different modality to that of the targets are perceived in cause retrieval of previous episodes and that this distractor-based retrieval decreases with increasing distractor dissimilarity following generalization processes as observed in Pavlovian conditioning.

#### Open Practices Statement

Data for both experiments is publicly available under 10.23668/psycharchives.2887. Code for analysis for both experiments is publicly available under 10.23668/psycharchives.2888. None of the experiments were preregistered.

## Footnotes

1 We used stimuli that appeared slightly larger than in Singh et al. (2016).

2 In Singh et al. (2016), this response mapping was reversed for half the participants, that is, half of the participants were instructed to respond to a diamond or a triangle by pressing the J-key, and to a cross and square by pressing the F-key. As we did not expect that the interaction hinges on this aspect, we did not incorporate a second response mapping.

3 For example, the DRB-effect for DR with two steps deviation is calculated as: DRB with two steps deviation = (RRDC-RRDR with two steps deviation)-(RCDC-RCDR with two steps deviation).

4 In our experimental design, a response repetition could occur with or without a target repetition (see, e.g., Frings et al., 2007; Giesen & Rothermund, 2014). To ensure that repeating or changing a target in response repetitions did not modulate the observed data pattern, we calculated the DRB-effects separately for response repetitions with target repetition and response repetitions without target repetition and included target relation (repetition vs. change) as a factor to the repeated-measures MANOVA on DRB effects computed for probe reaction times. Neither the main effect of target relation, *F*(1, 30) = 0.02, *p* = .889, ${\upeta}_p^2$ < .01, nor the interaction of target relation x strength of distractor deviation, *F*(3, 28) = 0.62, *p* = .608, ${\upeta}_p^2$ = .06, were significant, suggesting no modulating factor of a target repeating or not repeating in response repetitions on the effects of interest.

5 Repeating or changing the target in response repetitions modulated neither the overall DRB effects computed for probe error rates (main effect of target relation: *F*(1, 30) = 0.51, *p* = .479, ${\upeta}_p^2$ = .02) nor the decrease in DRB effects with increasing dissimilarity (interaction of target relation x strength of distractor deviation: *F*(3, 28) = 0.76, *p* = .529, ${\upeta}_p^2$ = .08).

6 Repeating or changing the target in response repetitions did not modulate the DRB effects with decreasing similarity computed for probe reaction times (main effect of target relation: *F*(1, 31) = 0.53, *p* = .473, ${\upeta}_p^2$ = .02; interaction of target relation x strength of distractor deviation: *F*(3, 29) = 0.48, *p* = .699, ${\upeta}_p^2$ = .05).

7 Repeating or changing the target in response repetitions did not modulate the DRB effects with decreasing similarity computed for probe error rates (main effect of target relation: *F*(1, 31) = 0.11, *p* = .748, ${\upeta}_p^2$ < .01; interaction of target relation x strength of distractor deviation: *F*(3, 29) = 0.89, *p* = .460, ${\upeta}_p^2$ = .08).

8 We would like to thank an anonymous reviewer for pointing this out.

9 Note, however, that DRB effects were also observed for response repetitions without target repetition, suggesting that a prime or probe display is more than a compound of a target feature and a distractor, but rather a combination of feature, distractor, and response given to it. However, we cannot rule out that response repetitions with target repetition might have benefited from the retrieval of audio-visual compounds.

10 In Experiment 1 of Mondor et al. (2003), trials could be identical, equivalent, or different from prime to probe, with two frequencies mapped on one key, and two frequencies mapped on the other key. Note that this is comparable to the task used in our Experiment 2. However, in our second study, (1) targets were accompanied by visual distractors and (2) the factor of response repetition with target repetition or without target repetition did not modulate the observed binding effects.
